# Prognostic role of minute ventilation/carbon dioxide production slope for perioperative morbidity and long-term survival in resectable patients with nonsmall-cell lung cancer: a prospective study using propensity score overlap weighting

**DOI:** 10.1097/JS9.0000000000000509

**Published:** 2023-05-18

**Authors:** Yaoshan Dun, Shaoping Wu, Ni Cui, Randal J. Thomas, Ray W. Squires, Thomas P. Olson, Karl P. Sylvester, Siqian Fu, Chunfang Zhang, Yang Gao, Yang Du, Ning Xu, Suixin Liu

**Affiliations:** a Department of Physical Medicine and Rehabilitation, Division of Cardiac Rehabilitation; b National Clinical Research Center for Geriatric Disorders; c Department of Thoracic Surgery; d Department of Neurology, Xiangya Hospital of Central South University, Changsha, Hunan; e Department of Data Analytics and Application, Ping An Technology, Shanghai, China; f Lung Function Unit, Cambridge University Hospitals NHSFT; g Respiratory Physiology, Royal Papworth Hospital NHSFT, Cambridge; h School of Cardiovascular and Metabolic Medicine & Sciences, Faculty of Life Sciences and Medicine, King’s College London, London, UK; i Division of Preventive Cardiology, Department of Cardiovascular Medicine, Mayo Clinic, Rochester, Minnesota, USA

**Keywords:** cardiopulmonary exercise testing, lung resection, minute ventilation/carbon dioxide production slope, nonsmall-cell lung cancer, overall survival, perioperative morbidity, relapse-free survival

## Abstract

**Background:**

The role of minute ventilation/carbon dioxide production ( $\dot{V}E$ / $\dot{V}$ CO_2_) slope, a ventilation efficiency marker, in predicting short-term and long-term health outcomes for patients with nonsmall-cell lung cancer (NSCLC) undergoing lung resection has not been well investigated.

**Material and Methods:**

This prospective cohort study consecutively enrolled NSCLC patients who underwent a presurgical cardiopulmonary exercise test from November 2014 to December 2019. The association of $\dot{V}E$ / $\dot{V}$ CO_2_ slope with relapse-free survival (RFS), overall survival (OS), and perioperative mortality was evaluated using the Cox proportional hazards and logistic models. Covariates were adjusted using propensity score overlap weighting. The optimal cut-off point of the $\dot{V}$ E/ $\dot{V}$ CO_2_ slope was estimated using the receiver operating characteristics curve. Internal validation was completed through bootstrap resampling.

**Results:**

A cohort of 895 patients [median age (interquartile range), 59 (13) years; 62.5% male] was followed for a median of 40 (range, 1–85) months. Throughout the study, there were 247 relapses or deaths and 156 perioperative complications. The incidence rates per 1000 person-years for relapses or deaths were 108.8 and 79.6 among patients with high and low $\dot{V}$ E/ $\dot{V}$ CO_2_ slopes, respectively [weighted incidence rate difference per 1000 person-years, 29.21 (95% CI, 7.30–51.12)]. A $\dot{V}$ E/ $\dot{V}$ CO_2_ slope of greater than or equal to 31 was associated with shorter RFS [hazard ratio for relapse or death, 1.38 (95% CI, 1.02–1.88), *P*=0.04] and poorer OS [hazard ratio for death, 1.69 (1.15–2.48), *P*=0.02] compared to a lower $\dot{V}E$ / $\dot{V}$ CO_2_ slope. A high $\dot{V}$ E/ $\dot{V}$ CO_2_ slope increased the risk of perioperative morbidity compared with a low $\dot{V}$ E/ $\dot{V}$ CO_2_ slope [odds ratio, 2.32 (1.54–3.49), *P*<0.001].

**Conclusions:**

In patients with operable NSCLC, a high $\dot{V}$ E/ $\dot{V}$ CO_2_ slope was significantly associated with elevated risks of poorer RFS, OS, and perioperative morbidity.

## Graphical abstract

**Figure:**
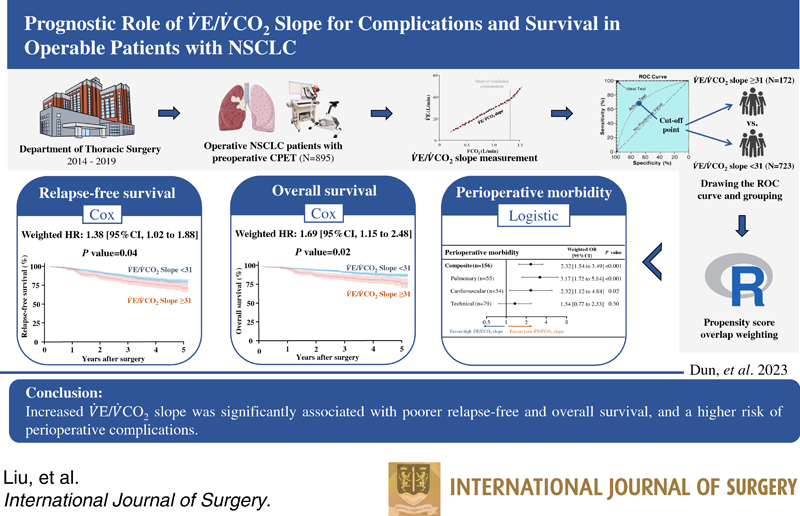

## Introduction

Highlights

- The increased minute ventilation/carbon dioxide production slope was significantly associated with poorer relapse-free and overall survival in patients with nonsmall-cell lung cancer who underwent lung resection.
- A high ventilation/carbon dioxide production slope was significantly related to a higher risk of perioperative morbidity in operable nonsmall-cell lung cancer patients.

Lung cancer is a primary contributor to global cancer-related mortality, with 5-year survival rates ranging from 26 to 64% ^1^. When it comes to early-stage nonsmall-cell lung cancer (NSCLC), which comprises approximately about 80–85% of lung cancers, lung resection remains the most effective treatment for achieving a cure^2^. However, this procedure is associated with a relatively high risk of perioperative morbidity, with an estimated incidence rate of 20–40%^3^. In addition, a significant number of patients with NSCLC experience relapse within 5 years after undergoing surgery, with relapse rates ranging from 30 to 55%^4^. The median survival time for patients who experience NSCLC relapse is ~21 months^5^. Therefore, it is essential to identify patients who are at high risk of perioperative complications, relapse, and mortality after lung resection to provide optimal patient-specific care.

Currently, cardiopulmonary exercise testing (CPET) is extensively used for presurgical assessment and complication risk stratification for thoracic surgery^6^. Among the CPET parameters, peak oxygen consumption is well-documented as a predictor of morbidity and mortality for patients undergoing lung surgery^7–9^. Another vital CPET parameter that has gained attention is the minute ventilation/carbon dioxide production ( $\dot{V}$ E/ $\dot{V}$ CO_2_) slope, which is a measure of ventilation efficiency. Several studies^10–16^ have investigated the prognostic value of $\dot{V}$ E/ $\dot{V}$ CO_2_ slope in postoperative complications after lung resection. However, the populations and cut-off points in these studies have varied.

The $\dot{V}$ E/ $\dot{V}$ CO_2_ slope has been found to have a prognostic role in predicting long-term mortality in patients with chronic heart failure^17,18^, chronic obstructive pulmonary disease (COPD)^19^, and pulmonary hypertension^20^ in recent studies. However, for patients with NSCLC, only two studies with relatively small sample sizes reported inconsistent results regarding the association between the $\dot{V}$ E/ $\dot{V}$ CO_2_ slope and overall survival (OS) after lung surgery. Furthermore, to the best of our knowledge, no study has reported the relationship between $\dot{V}$ E/ $\dot{V}$ CO_2_ slope and relapse-free survival (RFS).

This prospective observational study aimed to explore the prognostic value of the $\dot{V}$ E/ $\dot{V}$ CO_2_ slope for RFS and OS and to verify its effectiveness in predicting perioperative morbidity in NSCLC patients who underwent lung resection. The findings of this study might offer clinicians valuable evidence to determine whether the $\dot{V}$ E/ $\dot{V}$ CO_2_ slope should be incorporated into the management of patients with NSCLC undergoing lung resection.

## Materials and methods

The Ethics Committee approved this study (approval number 202010145). The need for informed consent was waived due to the study’s observational design. Patient information was anonymized to protect confidentiality. The study was registered with the Chinese Clinical Trial Registry. The work has been reported in line with the Strengthening the Reporting of Cohort Studies in Surgery (STROCSS) criteria^21^, Supplemental Digital Content 1, http://links.lww.com/JS9/A508.

### Participants

**Figure 1:**
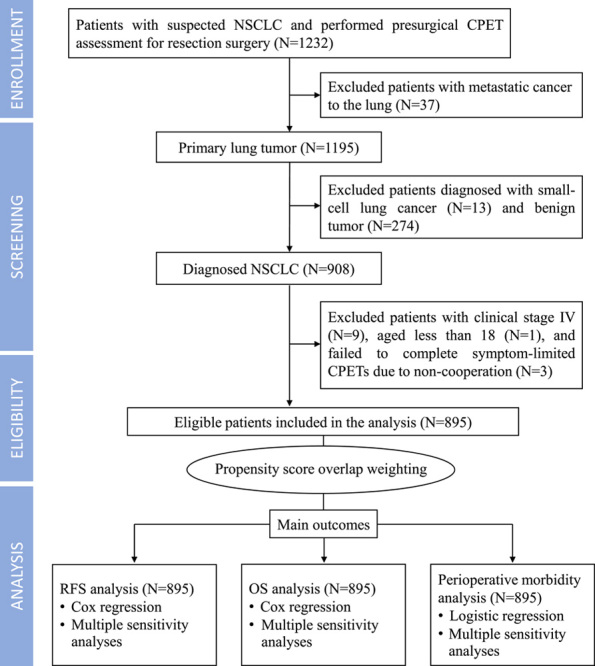
Flowchart from enrollment to analysis. CPET, cardiopulmonary exercise testing; NSCLC, nonsmall-cell lung cancer; RFS, relapse-free survival; OS, overall survival.

This prospective, observational study is a part of the Xiangya Hospital Exercise Testing project^22,23^. This study included consecutive patients with suspected NSCLC who were scheduled for lung resection and underwent a preoperative CPET within 1 week before the surgery between 1 November 2014, and 31 December 2019. All participants were referred for lung cancer surgery in line with the current clinical practice guidelines^24^ during the Multidisciplinary Tumor Board meeting. Patients aged less than 18 years or those diagnosed with small cell lung cancer, benign tumor, or distant metastasis were excluded from the study. Additionally, patients who failed to complete a symptom-limited CPET were excluded. Figure 1 shows the inclusion and exclusion flowcharts.

### Perioperative management

The same team of anaesthesiologists and board-certified thoracic surgeons performed the surgical procedures and provided postoperative care to the patients. Extubation was performed in the operating theater, followed by a 24 h stay in the post-anesthesia recovery room before transfer to the thoracic ward. The postoperative management protocol was standardized, with emphasis on early feeding, vigilant fluid balance, active mobilization, performing lung expansion exercises, and administering multimodal analgesia.

### The $\dot{V}$ E/ $\dot{V}$ CO_2_ slope

The patients underwent a symptom-limited CPET with a respiratory gas exchange measurement system, using a cycle ergometer with ramp protocol (CARDIOVIT system), to obtain the $\dot{V}$ E/ $\dot{V}$ CO_2_ slope. Standard exercise testing procedures were followed during all CPETs^22,23^. The $\dot{V}$ E/ $\dot{V}$ CO_2_ slope indicates pulmonary gas exchange efficiency and the ventilation demand to remove a certain amount of carbon dioxide produced by active tissues^25^. The method used to identify and validate the $\dot{V}$ E/ $\dot{V}$ CO_2_ slope is shown in eFigure 1 (Supplement, Supplemental Digital Content 2, http://links.lww.com/JS9/A509). The optimal cut-off point of the $\dot{V}$ E/ $\dot{V}$ CO_2_ slope for the outcomes was determined by receiver operating characteristics (ROC) analysis and the Youden index^26^. The ROC curves are provided in eFigure 2 (Supplement, Supplemental Digital Content 2, http://links.lww.com/JS9/A509). Patients with $\dot{V}$ E/ $\dot{V}$ CO_2_ slopes equal to or more than the cut-off point were categorized into the exposed group.

### RFS

The primary endpoint, RFS, referred to the time from enrollment (the day of the operation) to relapse, which was defined as cancer recurrence within or immediately adjacent to the treated area, mediastinal recurrence, distant recurrence, or death before 31 December 2021. Independent sources/methods, including the Residence Registration Office, the electronic medical record system, and contact with the participants’ families, were used to verify relapse and deaths. Follow-up was conducted in accordance with the follow-up guideline^27^, with physical examination and chest computed tomography every 3–6 months for the first 2 years after surgery and annually thereafter.

### OS and perioperative morbidity

The secondary outcomes were OS and perioperative morbidity. The follow-up duration for OS was from enrollment until death from any cause until the study’s censor date of 31 December 2021.

Perioperative morbidity was determined by the occurrence of respiratory, cardiovascular, and technical complications during hospitalization, deaths occurred before/within 30 days of hospital discharge, and composite complications.

### Covariates

At the time of enrollment, participant’s age, birth sex, standing height, weight, and smoking history were recorded. Information regarding histology, clinical stages, tumor node metastasis (TNM) stage, types of lung resection, and factors potentially related to the risk of RFS, OS, and perioperative morbidity were documented in the electronic medical record. Tumor staging was evaluated based on the guidelines of the American Joint Committee on Cancer TNM Staging Manual, Eighth Edition^28^. The accuracy of the data was verified through cross-checking.

### Sample size

A minimum of 10 equivalent events of RFS, OS, or perioperative morbidity for each adjusted covariate is recommended to ensure the proposed model’s accuracy^29^. In the present study, there were observed relapse or death events (*n*=247) and deaths (*n*=142), which exceeded the suggested range of 14–24 adjusted covariates. Thus, these observed events are adequate for developing the regression models using the propensity score overlap weighting technique, where all covariates are weighted and combined as a single regression covariate.

### Statistical analysis

The Shapiro–Wilk test was performed to determine normality for continuous variables. Descriptive statistics are presented as mean±SD for normally distributed continuous variables while as median interquartile range (IQR) for those with a non-normal distribution. Categorical variables are reported as counts (percentages). Standardized mean difference was employed as a measure of balance for individuals covariates before and after propensity score weighting^30^. Conventionally, an standardized mean difference less than 0.1 is considered adequate for balance^31,32^.

To balance covariates and minimize the effect of extreme propensities on model output, a propensity score model with covariates was established using the overlap weighting method^33^. Overlap weighting assigned weights proportionate to their probability of belonging to the alternative group^33,34^.

The Kaplan–Meier method was used for time-to-event analyses and compared with the two-sided log-rank test. Cox proportional hazards models were fitted with weights derived from overlap weighting to evaluate the association between the $\dot{V}$ E/ $\dot{V}$ CO_2_ slope and RFS and OS. To examine the association between the $\dot{V}$ E/ $\dot{V}$ CO_2_ slope with all-cause RFS or OS within a short time frame, the hazard ratios (HR) were calculated at 1-year intervals after enrollment. In addition, the crude and weighted incidence rates were computed as the relapse or mortality rate/1000 person-years^34^.

A logistic model with propensity score overlap weighting was fitted to explore the relationship between the $\dot{V}$ E/ $\dot{V}$ CO_2_ slope and perioperative morbidity. Crude and weighted odds ratios (OR) were estimated, along with the incidence rates as the perioperative morbidity rate/1000 surgeries.

Bootstrapping was used to assess the predictive effectiveness for internal validation, as this is an efficient validation method for all aspects of predictive model development^35,36^. Hence, 1000 bootstrap samples were generated, and the C-index and Brier scores were estimated. The Brier scores were used to assess the overall performance, ranging between 0 (perfect accuracy) and 1 (perfect inaccuracy)^37^. The consistency between the observed and predicted hazards of results was evaluated by the calibration slope^37^. A value close to 1 indicated a good overall agreement. The efficiency of the model was measured using the C-index to distinguish patients at high and low risks of outcomes^37^, with a value of 0.5 indicating no discrimination and a value of 1 indicating the best discrimination.

Interaction test were performed for all covariates (measured confounders), while the E-value was applied to assess the unmeasured confounders. The E-value is a statistical measure that quantifies the minimum strength of association that an unmeasured confounder would need to have with both the exposure and the outcome in order to explain away the observed association between the exposure and outcome^38^.

Prespecified subgroup analyses were conducted based on birth sex and age. Additional subgroups were determined by the significant factors in interaction tests. In the subgroup analyses, overlap weighting propensity scores were recreated before subgroup model development. Due to the potential for type I error resulting from multiple comparisons, subgroup analyses were considered exploratory.

All analyses were performed using R (version 4.2.0) software. The R statistical packages^39–46^ and code for the statistical analyses have been made publicly available on the GitHub repository at https://github.com/YSDun/VE-VCO2/blob/main/VE_VCO2.Rmd. Statistical significance was set at *P*<0.05 (two-sided).

## Results

### Participants characteristics

A total of 1232 patients suspected of having NSCLC underwent presurgical CPETs between 1 November 2014 and 31 December 2019. After a screening process, 895 were eligible and included in the study, while 337 were excluded due to benign tumors (*n*=274), metastatic lung cancer (*n*=37), small cell lung cancer (*n*=13), stage IV cancer (*n*=9), refusal to undergo CPETs (*n*=3), and age below 18 years old (*n*=1) (refer to Fig. 1). The median age of the 895 participants was 59 years (IQR, 13 years), and the majority of the participants were male (62.5%). All participants completed the symptom-limited CPETs. The characteristics of the CPET data are shown in eTable 1 (Supplement, Supplemental Digital Content 2, http://links.lww.com/JS9/A509). The ROC analysis identified a $\dot{V}$ E/ $\dot{V}$ CO_2_ greater than or equal to 31 as the optimal prognostic cut-off value in this study, as depicted by the ROC curves in eFigure 2 (Supplement, Supplemental Digital Content 2, http://links.lww.com/JS9/A509). Among the 895 participants, 172 (19.22%) had a $\dot{V}$ E/ $\dot{V}$ CO_2_ of greater than or equal to 31, while 723 (80.78%) had a $\dot{V}$ E/ $\dot{V}$ CO_2_ of less than 31. The mean $\dot{V}$ E/ $\dot{V}$ CO_2_ was 27.6 (SD, 4.6). Male participants, older individuals, those with a higher T stage, and those with a history of smoking, hypertension, and COPD were more likely to have a high $\dot{V}$ E/ $\dot{V}$ CO_2_. Further details are presented in Table 1. The Love plot, which illustrates the balance of covariates between high and low $\dot{V}$ E/ $\dot{V}$ CO_2_ groups before and after applying propensity score overlap weighting, can be found in eFigure 3 (Supplement, Supplemental Digital Content 2, http://links.lww.com/JS9/A509).

**Table 1:** Demographic data of the included 895 patients with NSCLC grouped based on $\dot{V}$ E/ $\dot{V}$ CO_2_ slope greater than or equal to 31 and less than 31, before and after applying propensity score overlap weighting.

	Crude			After propensity score overlap weighting		
Variable	$\dot{V}$ E/ $\dot{V}$ CO_2_ slope ≥31 (*N*=172)	$\dot{V}$ E/ $\dot{V}$ CO_2_ slope <31 (*N*=723)	Standardized mean difference	$\dot{V}$ E/ $\dot{V}$ CO_2_ slope ≥31 (*N*=122)	$\dot{V}$ E/ $\dot{V}$ CO_2_ slope <31 (*N*=122)	Standardized mean difference
Male,*n*(%)	123 (71.5)	436 (60.3)	0.238	83 (68.0)	83 (68.0)	<0.001
Age, mean±SD, years	63±8	57±9	0.647	61±8	61±8	<0.001
Height, mean±SD, cm	163.2±7.4	162.6±7.6	0.084	163.0±7.3	163.0±7.6	<0.001
Weight, mean±SD, kg	62.1±10.2	62.6±9.9	0.052	62.3±10.6	62.3±9.6	<0.001
Smoke ever,*n*(%)	92 (53.5)	308 (42.6)	0.219	61 (50.0)	61 (50.0)	<0.001
Histology,*n*(%)			0.192			<0.001
Adenocarcinoma	113 (65.7)	525 (72.6)		83 (68.0)	83 (68.0)	
Squamous cell	52 (30.2)	159 (22.0)		34 (27.9)	34 (27.9)	
Other NSCLCs	7 (4.1)	39 (5.4)		5 (4.1)	5 (4.1)	
Type of lung resections,*n*(%)			0.120			<0.001
Pneumonectomy	7 (4.1)	27 (3.7)		6 (4.9)	6 (4.9)	
Lobectomy	152 (88.4)	653 (90.3)		107 (87.7)	107 (87.7)	
Segmentectomy	3 (1.7)	13 (1.8)		2 (1.6)	2 (1.6)	
Wedge resection	3 (1.7)	4 (0.6)		1 (0.8)	1 (0.8)	
Explorative thoracotomy without lung resection	1 (0.6)	5 (0.7)		1 (0.8)	1 (0.8)	
Two lung lobes	6 (3.5)	21 (2.9)		5 (4.1)	5 (4.1)	
T stage,*n*(%)			0.216			<0.001
Tis	5 (2.9)	14 (1.9)		4 (3.3)	4 (3.3)	
T1	93 (54.1)	434 (60.0)		67 (54.9)	67 (54.9)	
T2	43 (25.0)	195 (27.0)		32 (26.2)	32 (26.2)	
T3	18 (10.5)	50 (6.9)		11 (9.0)	11 (9.0)	
T4	13 (7.6)	30 (4.1)		8 (6.6)	8 (6.6)	
N stage,*n*(%)			0.153			<0.001
N0	125 (72.7)	556 (76.9)		90 (73.8)	90 (73.8)	
N1	16 (9.3)	39 (5.4)		10 (8.2)	10 (8.2)	
N2 or N3	31 (18.0)	128 (17.7)		22 (18.0)	22 (18.0)	
M stage,*n*(%)						
M0	172 (100.0)	723 (100.0)	<0.001	122 (100.0)	122 (100.0)	<0.001
Medical history,*n*(%)						
Hypertension	54 (31.4)	139 (19.2)	0.283	34 (27.9)	34 (27.9)	<0.001
Dyslipidemia	13 (7.6)	76 (10.5)	0.103	10 (8.2)	10 (8.2)	<0.001
Diabetes mellitus	18 (10.5)	59 (8.2)	0.079	12 (9.8)	12 (9.8)	<0.001
CAD	32 (18.6)	142 (19.6)	0.026	23 (18.9)	23 (18.9)	<0.001
Cerebrovascular disease	12 (7.0)	7 (1.0)	0.311	4 (3.3)	4 (3.3)	<0.001
Tuberculosis	13 (7.6)	30 (4.1)	0.146	8 (6.6)	8 (6.6)	<0.001
Chronic bronchitis	15 (8.7)	58 (8.0)	0.025	11 (9.0)	11 (9.0)	<0.001
Emphysema	23 (13.4)	53 (7.3)	0.199	15 (12.3)	15 (12.3)	<0.001
COPD	12 (7.0)	14 (1.9)	0.246	5 (4.1)	5 (4.1)	<0.001

CAD, coronary artery disease; COPD, chronic obstructive pulmonary disease; NSCLC, nonsmall-cell lung cancer; $\dot{V}$ E/ $\dot{V}$ CO_2_, minute ventilation/carbon dioxide production. Continuous variables with normal distribution are expressed as mean±SD, and categorical variables are presented as numbers (percentages). Conventionally, the standardized mean difference of ≤0.1 is interpreted as a negligible difference in the mean of a covariate between groups.

### The association between the $\dot{V}$ E/ $\dot{V}$ CO_2_ slope and long-term RFS

**Figure 2:**
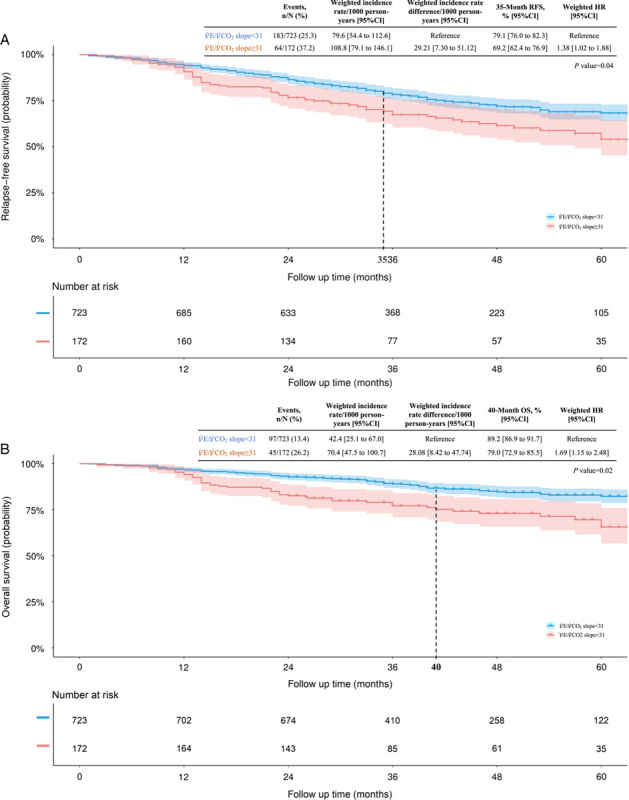
Kaplan–Meier estimates of A) relapse-free survival and B) overall survival in the NSCLC patients with $\dot{V}$ E/ $\dot{V}$ CO_2_ ≥31 versus $\dot{V}$ E/ $\dot{V}$ CO_2_ <31. NSCLC, nonsmall-cell lung cancer; $\dot{V}$ E/ $\dot{V}$ CO_2_, minute ventilation/carbon dioxide production. Dashed vertical lines at 35 months in Figure a) and 40 months in Figure b) indicate the median follow-up periods for relapse-free survival and overall survival, respectively. The shadow, along with the curves, represents the 95% CI.

During the study period, a total of 247 relapse and death events were observed. Upon implementing propensity score overlap weighting, the incidence rates of relapses or deaths per 1000 person-years were 108.8 in the high $\dot{V}$ E/ $\dot{V}$ CO_2_ slope group and 79.6 in the low $\dot{V}$ E/ $\dot{V}$ CO_2_ slope group, resulting in a weighted incidence rate difference (IRD) of 29.21 per 1000 person-years (95% CI, 7.30–51.12). A $\dot{V}$ E/ $\dot{V}$ CO_2_ slope of greater than or equal to 31 was associated with a high risk of shorter RFS compared to a lower $\dot{V}E$ / $\dot{V}$ CO_2_ slope (weighted HR for relapse or death, 1.38 [95% CI, 1.02–1.88]) (Fig. 2).

Several sensitivity analyses were conducted to evaluate potential sources of bias that might impact the observed association between the $\dot{V}$ E/ $\dot{V}$ CO_2_ slope and RFS. First, interaction tests were tested to examine the role of measured covariates. No significant interaction between measured covariates and the association between the $\dot{V}$ E/ $\dot{V}$ CO_2_ slope and RFS was found (all *P*>0.05) (Supplement eTable 2, Supplemental Digital Content 2, http://links.lww.com/JS9/A509). Prespecified subgroup analyses were also conducted, revealing that the relationship between the $\dot{V}$ E/ $\dot{V}$ CO_2_ slope and RFS remained statistically significant across male, without cerebrovascular, and without tuberculosis subgroups (Supplement eFigure 4, Supplemental Digital Content 2, http://links.lww.com/JS9/A509). Furthermore, we examined the impact of the duration of follow-up by plotting HRs at yearly intervals following the index date. The results demonstrated that a high $\dot{V}$ E/ $\dot{V}$ CO_2_ slope was associated with poorer RFS within the last three 1-year intervals (Supplement eFigure 5, Supplemental Digital Content 2, http://links.lww.com/JS9/A509). Moreover, the study employed the E-value test to evaluate the potential for bias resulting from unmeasured confounders. The calculated E-value was 1.81, which exceeds the HRs of most recognized risk factors, such as smoking (HR=1.41), for lung cancer prognosis as reported in previous literature^47^, indicating the observed association between the $\dot{V}$ E/ $\dot{V}$ CO_2_ slope and RFS is less likely to be explained by an unmeasured confounder (eTable 3). In addition, we conducted an internal validation using bootstrapping resampling to assess the robustness of the observed association. The analyses showed that the Brier score was 0.18 (95% CI, 0.16–0.19), the calibration slope was 0.82 (95% CI, 0.79–0.84), and the C-index was 0.61 (95% CI, 0.55–0.67). These results indicate the good overall performance of the regression model on the association between the $\dot{V}$ E/ $\dot{V}$ CO_2_ slope and RFS^37^.

### The association between the $\dot{V}$ E/ $\dot{V}$ CO_2_ slope and long-term OS

During the study period, a total of 142 patients died, with death rates of 70.4 and 42.4 deaths per 1000 person-years among patients with high and low $\dot{V}$ E/ $\dot{V}$ CO_2_ slopes, respectively. The weighted IRD in terms of death between the two groups was 28.08 (95% CI, 8.42 to 47.74) per 1000 person-years. A $\dot{V}$ E/ $\dot{V}$ CO_2_ slope of greater than or equal to 31 was significantly associated with a high risk of poorer OS compared to a lower $\dot{V}E$ / $\dot{V}$ CO_2_ slope [HR for death, 1.69 (95% CI, 1.15–2.48)] (Fig. 2).

Multiple sensitivity analyses were conducted to evaluate potential sources of bias that could affect the observed association between the $\dot{V}$ E/ $\dot{V}$ CO_2_ slope and OS. In testing for interactions, only the ‘smoking ever’ exhibited a significant interaction with the observed association between the $\dot{V}$ E/ $\dot{V}$ CO_2_ slope and OS (Supplement eTable 2, Supplemental Digital Content 2, http://links.lww.com/JS9/A509). In subgroup analyses, we found that the relationship between the $\dot{V}$ E/ $\dot{V}$ CO_2_ slope and OS remained statistically significant across male, elder, and smoking ever subgroups (Supplement eFigure 4 http://links.lww.com/JS9/A509). When examining the impact of follow-up duration, the HRs plotted at 1-year intervals following the index date indicated that a high $\dot{V}$ E/ $\dot{V}$ CO_2_ slope was significantly associated with poorer OS within the last four 1-year intervals (eFigure 5). The calculated E-value was 2.23, which exceeds the HRs of most recognized risk factors, such as histologic grade (HR=1.83), for survival in patients with NSCLC as reported in previous literature^48^, indicating the observed association between the $\dot{V}$ E/ $\dot{V}$ CO_2_ slope and OS is less likely to be explained by an unmeasured confounder. Internal validation of the association between the $\dot{V}$ E/ $\dot{V}$ CO_2_ slope and OS was conducted, yielding a Brier score of 0.13 (95% CI, 0.11–0.15), a calibration slope of 0.79 (95% CI, 0.75–0.83), and a C-index of 0.62 (95% CI, 0.51–0.75). These findings indicate the good overall performance of the regression model on the association between the $\dot{V}$ E/ $\dot{V}$ CO_2_ slope and OS.

### The association between the $\dot{V}$ E/ $\dot{V}$ CO_2_ slope and perioperative morbidity

Among the 895 participants, 156 experienced a total of 168 perioperative complications, which included 55 pulmonary, 34 cardiovascular, and 79 surgical technique-related complications. There were 291.7 and 150.8 complications per 1000 surgeries among patients with high and low $\dot{V}$ E/ $\dot{V}$ CO_2_ slope, respectively [weighted IRD per 1000 surgeries, 140.85 (95% CI, 67.04–214.66)]. Participants with a $\dot{V}$ E/ $\dot{V}$ CO_2_ slope of greater than or equal to 31 had a 2.32 times higher risk of perioperative complications than those with a $\dot{V}$ E/ $\dot{V}$ CO_2_ of less than 31, with a weighted OR of 2.32 (95% CI, 1.54–3.49). Table 2 presents the incidence rates and corresponding ORs for pulmonary, cardiovascular, and surgical technique-related complications.

**Table 2:** Association of the $\dot{V}$ E/ $\dot{V}$ CO_2_ Slope with perioperative morbidity in the included 895 patients with NSCLC after applying propensity score overlap weighting.

	$\dot{V}$ E/ $\dot{V}$ CO_2_ slope ≥31 (*N*=172)	$\dot{V}$ E/ $\dot{V}$ CO_2_ slope <31 (*N*=723)			
Perioperative morbidity	Weighted incidence rate/1000 surgeries		Weighted difference/1000 surgeries [95% CI] ^*b*^	Weighted OR [95% CI]	*P*
Composite (*n*=156) ^*a*^	291.7	150.8	140.85 [67.04–214.66]	2.32 [1.54–3.49]	<0.001
Pulmonary (*n*=55)	131.8	45.7	86.12 [33.79–138.45]	3.17 [1.72–5.84]	<0.001
Cardiovascular (*n*=34)	75.0	33.7	41.31 [1.07–81.55]	2.32 [1.12–4.84]	0.02
Technical (*n*=79)	115.3	88.5	26.80 [-25.90–79.50]	1.34 [0.77–2.33]	0.30

NSCLC, nonsmall-cell lung cancer; OR, odds ratio; $\dot{V}$ E/ $\dot{V}$ CO_2_, minute ventilation/carbon dioxide production. Respiratory complications included atelectasis, pulmonary embolism, pneumonia, respiratory failure, and acute respiratory distress syndrome. Cardiovascular complications included cardiac arrhythmias requiring drug therapy, acute coronary syndrome, cardiac failure, and stroke. Technical complications included chylothorax, prolonged lung air leakage, blood loss or massive hemothorax requiring blood transfusion, and wound and/or chest infections.

^*a*^For the composite analysis, only one event was included if a patient developed more than one perioperative morbidity.

Cerebrovascular disease, tuberculosis, COPD, and types of lung resection exhibited significant interactions with the observed association between the $\dot{V}$ E/ $\dot{V}$ CO_2_ slope and perioperative morbidity (Supplement eTable 2, Supplemental Digital Content 2, http://links.lww.com/JS9/A509). In subgroup analyses, the association between the $\dot{V}$ E/ $\dot{V}$ CO_2_ slope and perioperative morbidity remained statistically significant across male, female, smoking ever, and nonsmoking groups (Supplement eFigure 4, Supplemental Digital Content 2, http://links.lww.com/JS9/A509). The calculated E-value was 2.42, which exceeds the HRs of most recognized risk factors for perioperative morbidity in patients with NSCLC, indicating a lower likelihood of the observed association being explained by an unmeasured confounding variable. Internal validation revealed a Brier score of 0.16 (95% CI, 0.13–0.19), a calibration slope of 0.72 (95% CI, 0.70–0.74), and a C-index of 0.66 (95% CI, 0.54–0.78). These findings indicate that the regression model on the association between the $\dot{V}$ E/ $\dot{V}$ CO_2_ slope and perioperative morbidity demonstrated a good overall performance.

## Discussion

This study, for the first time, explored the relationship between the $\dot{V}$ E/ $\dot{V}$ CO_2_ slope and RFS in patients with NSCLC who underwent lung cancer resection. Additionally, this is the largest-scale and longest follow-up study conducted to date on the association of the $\dot{V}$ E/ $\dot{V}$ CO_2_ slope with OS and perioperative morbidity. Our findings indicate a significant association between the $\dot{V}$ E/ $\dot{V}$ CO_2_ slope and both RFS, OS, and perioperative morbidity. This suggests that the $\dot{V}$ E/ $\dot{V}$ CO_2_ slope may serve as a valuable tool for identifying patients with NSCLC who are at a higher risk of experiencing adverse outcomes.

There have been several controversies regarding the use of the $\dot{V}$ E/ $\dot{V}$ CO_2_ slope in the presurgical assessment of patients with NSCLC. While some studies have shown that the $\dot{V}$ E/ $\dot{V}$ CO_2_ slope is a useful prognostic marker for predicting patient prognosis, others have reported conflicting results. For example, Miyazaki *et al*.^49^ enrolled 172 patients undergoing lobectomy or segmentectomy, and found that the $\dot{V}$ E/ $\dot{V}$ CO_2_ slope was a significant predictor for 90-day and 2-year survival, whereas Bédat *et al*.^15^ reported that no significant association was observed between cancer-related death and the $\dot{V}$ E/ $\dot{V}$ CO_2_ slope. The current study, which features the largest sample size and the longest follow-up to date, supported that $\dot{V}$ E/ $\dot{V}$ CO_2_ slope is a valuable tool for predicting prognosis in patients with NSCLC undergoing lung resection. However, further research is warranted to validate the usefulness of the $\dot{V}$ E/ $\dot{V}$ CO_2_ slope in larger cohorts and across diverse clinical settings.

Moreover, there is a lack of consensus on what constitutes a normal value or optimal cut-off value for the $\dot{V}$ E/ $\dot{V}$ CO_2_ slope in patients with NSCLC. Previous studies have proposed varying normal values of $\dot{V}$ E/ $\dot{V}$ CO_2_ slope for the general population, ranging from 23 to 31, which might differ by sex and age (progressive increase with increasing age)^50^. Similarly, a study by Kato *et al*.^51^ identified different cut-off value for patients of different ages, with values ranging from 32 for patients aged less than or equal to 55 years and 35 for those aged 56–70 years with heart failure. In addition, two studies focusing on the patients undergoing lung resection reported different threshold values predicting perioperative morbidity: 34^10^ and 35^12^, respectively. In the present study of Asian NSCLC patients with a median age of 59 (IQR, 13) who underwent lung resection, we found that a $\dot{V}$ E/ $\dot{V}$ CO_2_ slope of greater than or equal to 31 was associated with a higher rate of relapse, all-cause mortality, and perioperative morbidity. These results suggest that the optimal cut-off value of the $\dot{V}$ E/ $\dot{V}$ CO_2_ slope might differ based on the populations and diseases prognoses. Therefore, further higher-quality studies involving diverse racial and ethnic populations, ages, and outcomes are required to explore the optimal predictive cut-off values of the $\dot{V}$ E/ $\dot{V}$ CO_2_ slope.

Several significant developments have recently occurred in using the $\dot{V}$ E/ $\dot{V}$ CO_2_ slope for clinical assessments. One notable advancement is the development of more sophisticated techniques, including advanced signal processing techniques and various validated methods, in analyzing the $\dot{V}$ E/ $\dot{V}$ CO_2_ slope in 2021^52^. Additionally, the extended use of $\dot{V}$ E/ $\dot{V}$ CO_2_ slope in presurgical assessment for other diseases, such as colorectal cancer, has been reported in 2019 and 2020^53,54^. Furthermore, a recent study published in 2023 suggested that the $\dot{V}$ E/ $\dot{V}$ CO_2_ slope is a valuable predictor of the composite endpoint of cardiovascular mortality and hospital admission in individuals with cardiovascular risk factors, adding predictive value to peak oxygen consumption^55^.

**Figure 3:**
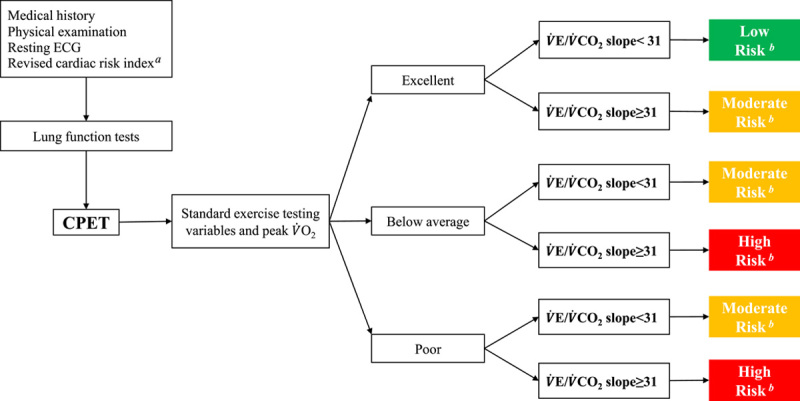
A suggested algorithm for using the $\dot{V}$ E/ $\dot{V}$ CO_2_ slope in the assessment of the perioperative morbidity risk and long-term prognosis in patients with resectable nonsmall cell lung cancer. CPET, cardiopulmonary exercise testing; ECG, electrocardiograph; peak $\dot{V}$ O_2_, peak oxygen consumption; $\dot{V}$ E/ $\dot{V}$ CO_2_, minute ventilation/carbon dioxide production. ^*a*^ Revised cardiac risk index [61]: (1) high risk surgery (including lobectomy or pneumonectomy); (2) ischemic heart disease (prior myocardial infarction, angina pectoris); (3) heart failure; (4) insulin-dependent diabetes; (5) previous stroke of transient ischemic attack; (6) creatinine ≥2 mg·dl^-1^. ^*b*^ Low risk in green indicates excellent prognosis and low risk for perioperative complications. Moderate risk in yellow and high risk in red suggest progressively worse prognosis and a higher risk of perioperative complications. Patients who are classified as having moderate and high risk warrant strong consideration of more aggressive medical management and surgical options.

A suggested algorithm for using the $\dot{V}$ E/ $\dot{V}$ CO_2_ slope in the assessment of perioperative morbidity risk and long-term prognosis in resectable patients with NSCLC is proposed based on recent achievements in the field of using the $\dot{V}$ E/ $\dot{V}$ CO_2_ slope in research and published clinical guidelines^56,57^ on the selection of patients with lung cancer for surgery (Fig. 3). Further studies on the prognostic value of using the $\dot{V}$ E/ $\dot{V}$ CO_2_ slope combined with other well-evidenced and emerging predictors, such as tumor characteristics and gene mutations^9,58,59^, in patients with NSCLC are needed.

The $\dot{V}$ E/ $\dot{V}$ CO_2_ slope, which measures the gas exchange efficiency during exercise, has been associated with patient prognosis in various diseases^25^. However, the underlying mechanisms remain poorly elucidated. In this study, we found that a higher $\dot{V}$ E/ $\dot{V}$ CO_2_ slope was associated with a poorer prognosis in patients with resectable NSCLC, likely due to two primary physiological mechanisms. First, an increased $\dot{V}$ E/ $\dot{V}$ CO_2_ slope reflects an increased ventilation requirement for a given amount of carbon dioxide production^25^, indicating ventilatory inefficiency. This could be caused by various factors, such as impaired lung function, reduced cardiac output, decreased diffusion capacity, increased breathing effort, pulmonary vascular disease, and anatomical abnormalities. Second, a higher $\dot{V}$ E/ $\dot{V}$ CO_2_ slope could also result from augmented carbon dioxide production along with an excessive increase in minute ventilation. Increased carbon dioxide production could occur due to various factors, such as increased metabolic demand or impaired energy utilization that are associated with cancer development and progression^60^. Overall, these physiological mechanisms highlight the complex relationship between gas exchange efficiency and prognosis in patients with resectable NSCLC.

We acknowledge several potential limitations associated with our study. First, since this was an observational study, despite the use of the propensity score overlap weighting to minimize bias, residual and unmeasured confounding might have persisted. Second, the lack of details on radiotherapy or chemoradiotherapy might have affected the long-term RFS and OS. However, we adjusted for the TNM staging, which determines the accessibility and eligibility of neoadjuvant radiotherapy and chemoradiotherapy. Finally, caution should be exercised when interpreting the subgroup results due to the limited number of events occurring in some subgroups.

## Conclusions

A high $\dot{V}$ E/ $\dot{V}$ CO_2_ slope was significantly associated with an elevated risk of poorer RFS, OS, and perioperative morbidity in operable patients with NSCLC.

## Ethical approval

Ethical approval for this study (approval number 202010145) was provided by the Ethical Committee of Xiangya Hospital, Changsha, China on 11 November 2020.

## Consent

As this observational study did not impact patient care, the Ethics Committee of the Xiangya Hospital waived the need for informed written consent (approval number 202010145). Patient information was anonymized to protect confidentiality.

## Conflicts of interest disclosure

All authors report no conflicts of interest relevant to this article.

## Sources of funding

This work is funded by the National Natural Science Foundation of China (82172549 to Suixin Liu, and 82272613 and 82002403 to Yaoshan Dun), the Natural Science Foundation of Hunan Province (2021JJ70073 to Suixin Liu and 2021JJ40981 to Yaoshan Dun). The funding sources were not involved in trial design, patient recruitment, data collection, analysis, interpretation or any aspect pertinent to the study. Funding information for this article has been deposited with the Crossref Funder Registry.

## Author contribution

Y.S.D.: conceptualization, methodology, formal analysis, writing - original draft, writing - review and editing, project administration, funding acquisition; S.W.: investigation, data curation, writing - original draft, writing - review and editing, visualization; N.C.: investigation, data curation, writing - original draft, writing - review and editing, visualization; R.J.T.: conceptualization, writing - original draft, writing - review and editing; R.W.S.: conceptualization, writing - original draft, writing - review and editing; Thomas P. Olson.: conceptualization, writing - original draft, writing - review and editing; K.P.S.: conceptualization, writing - original draft, writing - review and editing; S.F.: validation, investigation, data curation; C.Z.: investigation, resources, writing - review and editing; Y.G.: investigation, resources, writing - review and editing; Y.D.: methodology, writing - review and editing, visualization; N.X.: validation, software, formal analysis; S.L.: conceptualization, resources, writing - review and editing, supervision, funding acquisition. All authors contributed to the review and revision and have seen and approved the final version of the manuscript.

## Research registration unique identifying number (UIN)

1. Name of the registry: Chinese Clinical Trial Registry
2. Unique identifying number or registration ID: ChiCTR2100048120
3. Hyperlink to your specific registration (must be publicly accessible and will be checked): https://www.chictr.org.cn/searchprojEN.html?title=&officialname=&subjectid=&regstatus=&regno=ChiCTR2100048120&secondaryid=&applier=&studyleader=&createyear=&sponsor=&secsponsor=&sourceofspends=&studyailment=&studyailmentcode=&studytype=&studystage=&studydesign=&recruitmentstatus=&gender=&agreetosign=&measure=&country=&province=&city=&institution=&institutionlevel=&intercode=&ethicalcommitteesanction=&whetherpublic=&minstudyexecutetime=&maxstudyexecutetime=&btngo=btn

## Guarantor

Suixin Liu, Professor of Medicine, Xiangya Hospital of Central South University, Medical Director, Division of Cardiac Rehabilitation, Department of Physical Medicine & Rehabilitation, Xiangya Hospital of Central South University, Changsha, 410008, China. Tel: +86 731 8432 7179. Email: liusuixin@csu.edu.cn.

## Provenance and peer review

Not commissioned, externally peer-reviewed.

## Data availability statement

The research data belongs to the Cardiac Rehabilitation Center, Xiangya Hospital of Central South University, China. The R statistical packages^39–46^ and code for the statistical analyses have been made publicly available on the GitHub repository at https://github.com/YSDun/VE-VCO2/blob/main/VE_VCO2.Rmd. The corresponding author may, upon request, provide individual participant data as the basis for the results reported in this article after taking the necessary steps to ensure that no individual is identifiable.

## Provenance and peer review

Not commissioned, externally peer-reviewed.

## Supplementary Material

SUPPLEMENTARY MATERIAL
