# Supplementary material for: Prognostic role of minute ventilation/carbon dioxide production slope for perioperative morbidity and long-term survival in resectable patients with nonsmall-cell lung cancer: a prospective study using propensity score overlap weighting
Source: Int J Surg. 2023 May 18;109(9):2650–9. doi: 10.1097/JS9.0000000000000509 (PMC10498874; doi:10.1097/JS9.0000000000000509)
Supplement: SUPPLEMENTARY MATERIAL [file js9-109-2650-s002.docx]

**eTable 1.** Characteristics of Symptom-Limited Cardiopulmonary Exercise Tests.

| **Items** | **Number (%) /Mean**±**SD *^a^*** |
| --- | --- |
| **Exhaustion/Good effort** | 895 (100.0) |
| Achieved 85% of predicted HR (N, %) | 711 (79.4) |
| RPE ≥17 (N, %) | 761 (85.0) |
| RER ≥1.05 (N, %) | 100 (100.0) |
| RER ≥1.10 (N, %) | 842 (94.1) |
| Presence of plateau of HR or oxygen consumption (N, %) | 6 (0.7) |
| Presence of clinical symptoms (N, %) | 214 (23.9) |
| Dizziness | 150 (16.8) |
| Abnormal BP response | 50 (5.6) |
| Chest pain or distress | 22 (2.5) |
| Skeletomuscular limit | 9 (1.0) |
| ST segment elevation or depression ≥3 mm | 2 (0.2) |
|  |  |
| **CPET parameters** |  |
| $\dot{V}$E/$\dot{V}$CO_2_ slope (mean, SD) | 27.6±4.6 |
| Peak RER (mean, SD) | 1.09±0.11 |
| Peak workload, watts (mean, SD) | 102±29 |
| ECG (N, %) |  |
| Positive | 103 (11.5) |
| Negative | 657 (73.4) |
| Nondiagnostic | 135 (15.1) |
| Rest HR, bpm (mean, SD) | 86±14 |
| Peak HR, bpm (mean, SD) | 148±16 |
| Rest SBP, mmHg (mean, SD) | 128±17 |
| Rest DBP, mmHg (mean, SD) | 77±10 |
| Peak SBP, mmHg (mean, SD) | 185±24 |
| Peak DBP, mmHg (mean, SD) | 90±13 |
| Peak $\dot{V}$O_2_, ml/kg/min (mean, SD) | 22.9±4.5 |
| Breath reserve, % (mean, SD) | 52±15 |
| Peak respiratory frequency, breath/min (mean, SD) | 30±6 |

BP, blood pressure; DBP, diastolic blood pressure; ECG, electrocardiograph; HR, heart rate; peak $\dot{V}$O_2_, peak oxygen consumption; RER, respiratory exchange rate; RPE, Rating of Perceived Exertion; SD, standard deviation; SBP, systolic blood pressure; $\dot{V}$E/$\dot{V}$CO_2_, minute ventilation/carbon dioxide production.

***^a^*** Continuous data are presented as mean±SD, and categorical variables are shown as number and percentage.

**eTable 2.** The *P* values for Interaction between the $\dot{V}$E/$\dot{V}$CO_2_ Slope and Each Covariate in Predicting Relapse-free Survival, Overall Survival and Perioperative Morbidity.

| **Factors** | **Relapse-free survival** | **Overall**  **survival** | **Perioperative morbidity** |
| --- | --- | --- | --- |
| Sex | 0.24 | 0.03 | 0.39 |
| Age | 0.85 | 0.45 | 0.03 |
| Weight | 0.85 | 0.79 | 0.32 |
| Height | 0.50 | 0.16 | 0.99 |
| Smoke ever | 0.49 | 0.004 | 0.58 |
| Hypertension | 0.75 | 0.34 | 0.08 |
| Dyslipidemia | 0.36 | 0.19 | 0.27 |
| Diabetes mellitus | 0.95 | 0.11 | 0.58 |
| CAD | 0.89 | 0.60 | 0.63 |
| Cerebrovascular disease | 0.30 | 0.68 | <0.001 |
| Tuberculosis | 0.051 | 0.26 | <0.001 |
| Chronic bronchitis | 0.57 | 0.53 | 0.21 |
| Emphysema | 0.52 | 0.75 | 0.051 |
| COPD | 0.72 | 0.26 | 0.02 |
| Type of lung resections | 0.054 | 0.54 | 0.001 |
| Histology | 0.56 | 0.07 | 0.48 |
| T stage | 0.90 | 0.19 | 0.49 |
| N stage | 0.99 | 0.34 | 0.64 |

CAD, Coronary artery disease; COPD, chronic obstructive pulmonary disease, $\dot{V}$E/$\dot{V}$CO_2_, minute ventilation/carbon dioxide production.

**eTable 3.** E-values for Main Outcomes.

| Outcome | HR (OR)*^a^* | 95% CI | E-value | Lower limit of CI |
| --- | --- | --- | --- | --- |
| Relapse or death | 1.38 | 1.02 to 1.88 | 1.81 | 1.13 |
| Death | 1.69 | 1.15 to 2.48 | 2.23 | 1.44 |
| Perioperative morbidity | 2.32 | 1.54 to 3.49 | 2.42 | 1.79 |

*^a^* Hazard ratio (HR) for relapse-free and overall survivals; Odds ratio (OR) for perioperative morbidity. CI, confidence interval. The E-value is a metric as the minimum strength of association, on the HR or OR scale, that an unmeasured confounder would need to have with both the exposure and outcome to fully explain away the observed association between the exposure and outcome. An illustration of the E-value interpretation is as follows: we observe an E-value of 1.81 for the association between relapse or death and the $\dot{V}$E/$\dot{V}$CO_2_ slope. This implies that residual confounding could explain the observed association between relapse or death and the $\dot{V}$E/$\dot{V}$CO_2_ slope if there exists an unmeasured covariate having a relative risk association at least as large as 1.81 with both relapse or death and impaired the $\dot{V}$E/$\dot{V}$CO_2_ slope. Nonetheless, it is important to note that the majority of known risk factors for relapse or death or impaired $\dot{V}$E/$\dot{V}$CO_2_ slope in NSCLC patients have hazard ratios much lesser than 1.81, which indicates that the observed association between relapse or death and the $\dot{V}$E/$\dot{V}$CO_2_ slope was less likely to be explained by an unmeasured confounder. $\dot{V}$E/$\dot{V}$CO_2_, minute ventilation/carbon dioxide output.

**eFigure 1.** The Method to Identify and Verify the $\dot{V}$E/$\dot{V}$CO_2_ Slope. The $\dot{V}$E/$\dot{V}$CO_2_ slope represents the relationship between $\dot{V}$E, on the Y axis, and $\dot{V}$CO_2_, on the X axis, both in L/min. It was measured by linear regression and calculated from the initiation of exercise to the onset of ventilatory compensation (second ventilatory threshold), excluding the non-linear partial data after metabolic acidosis. The onset of ventilatory compensation (if present) was identified a) and b) on the point where $\dot{V}$E increased out of proportion to $\dot{V}$CO_2_, and was confirmed by c) establishing the nadir of the ventilatory equivalents for carbon dioxide versus exercise time (min) relationship and d) the downwards deflection point of end-tidal CO_2_ pressure (PetCO_2_) versus exercise time. $\dot{V}$E/$\dot{V}$CO_2_, minute ventilation/carbon dioxide output.

**eFigure 2.** The Receiver Operating Characteristics (ROC) Analysis of the $\dot{V}$E/$\dot{V}$CO_2_ Slope for a) Relapse-free Survival, b) Overall Survival and c) Perioperative Morbidity.

**eFigure 3.** Love Plot for Absolute Standardized Mean Differences before and after Propensity Score Overlap Weighting to Assessing the Balance in Covariates between Participants in $\dot{V}$E/$\dot{V}$CO_2_ Slope ≥31 and <31 Groups.

**eFigure 4.** Subgroup Analyses of the Association of the $\dot{V}$E/$\dot{V}$CO_2_ Slope to a) Relapse or Death, b) Death, and c) Perioperative Complication Events in NSCLC Patients with High $\dot{V}$E/$\dot{V}$CO_2_ Slope versus Low $\dot{V}$E/$\dot{V}$CO_2_ Slope.

NSCLC, nonsmall cell lung cancer; COPD, chronic obstructive pulmonary disease; HR, hazard ratio; $\dot{V}$E/$\dot{V}$CO_2_, minute ventilation/carbon dioxide output.

**eFigure 5.** Hazard Ratio (HR) for 1-5 Years for Relapse or Death and Death after the Index Date. $\dot{V}$E/$\dot{V}$CO_2_, minute ventilation/carbon dioxide output.
